# The Dichotomy in Degree Correlation of Biological Networks

**DOI:** 10.1371/journal.pone.0028322

**Published:** 2011-12-02

**Authors:** Dapeng Hao, Chuanxing Li

**Affiliations:** College of Bioinformatics Science and Technology, Harbin Medical University, Harbin, People's Republic of China; University of Michigan, United States of America

## Abstract

Most complex networks from different areas such as biology, sociology or technology, show a correlation on node degree where the possibility of a link between two nodes depends on their connectivity. It is widely believed that complex networks are either disassortative (links between hubs are systematically suppressed) or assortative (links between hubs are enhanced). In this paper, we analyze a variety of biological networks and find that they generally show a dichotomous degree correlation. We find that many properties of biological networks can be explained by this dichotomy in degree correlation, including the neighborhood connectivity, the sickle-shaped clustering coefficient distribution and the modularity structure. This dichotomy distinguishes biological networks from real disassortative networks or assortative networks such as the Internet and social networks. We suggest that the modular structure of networks accounts for the dichotomy in degree correlation and vice versa, shedding light on the source of modularity in biological networks. We further show that a robust and well connected network necessitates the dichotomy of degree correlation, suggestive of an evolutionary motivation for its existence. Finally, we suggest that a dichotomous degree correlation favors a centrally connected modular network, by which the integrity of network and specificity of modules might be reconciled.

## Introduction

Topological features of molecular networks have been studied extensively because of their relevance to the function and organization of living cells [1], [2]. A remarkable feature of most real networks is degree correlation, where the probability that two nodes are attached depends on their degrees [3], [4]. The importance of this stems from the fact that the structure of a network is highly determined by the correlation pattern of node's degrees. Examples of complex networks having different degree correlation patterns and very different network structures include the Internet, World Wide Web (WWW), collaboration relationships and metabolic networks [5]. The degree correlation pattern can be negative (*disassortative*), so that links between nodes with similar degree level are systematically suppressed, or positive (*assortative*). In particular, biological networks are believed to be disassortative, where a strong effective repulsion between highly connected nodes (hubs) increases the specificity of functional modules and stability of networks [4], [6], [7], [8].

**Figure 1:**
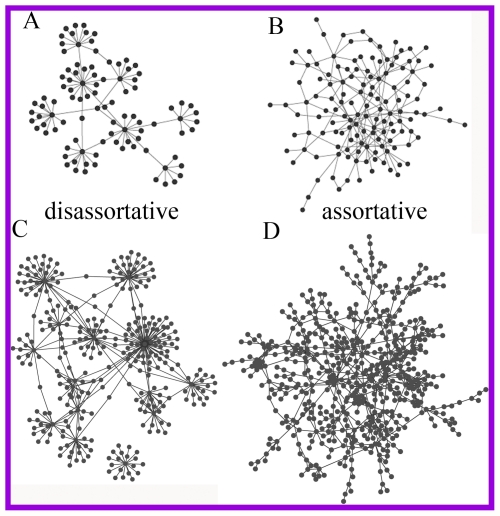
Disassortative and assortative networks. Schematic illustration of a disassortative network (**A**) and an assortative network (**B**). **C**. The 15 best connected proteins and their direct links to other proteins of yeast protein network constructed by proteins localized in nucleus. **D**. The rest of network after removal of the 15 best connected nodes. Nodes disconnected to the largest component are not shown. A predominant feature of **B** and **D** is the over-abundance of links between low connected nodes.

From a purely topological perspective, disassortative and assortative networks are highly different. A schematic illustration of a disassortative network and an assortative network is shown in Figure 1A and 1B. The differences between them are clear to see. The disassortative network is spread by the repulsion of hubs, suggestive of a picture of modularity with nodes organized around dispersed hubs [4]. The assortative network, on the contrary, is integrated by fully connected hubs. It is found that disassortativity produces better connected but vulnerable networks, whereas assortativity gives rise to less connected but resilient networks [3], [6]. In this paper, we show that, biological networks of a living cell have a better degree correlation pattern, which gives them the advantages of both disassortativity and assortativity, and enables them to avoid the disadvantages.

## Results

### Degree correlation of yeast protein interaction network in nucleus

To illustrate the degree correlation pattern in biological networks, we consider a representative network of yeast protein interaction and found that is neither disassortative nor assortative. According to a high-confidence (HC) dataset of yeast physical protein interactions [9], we abstract a small part of the protein network formed by proteins localized in nucleus, with 890 nodes and 1399 links. To quantify the degree correlation, we use a measure known as assortative coefficient, *r*, which is *Pearson coefficient* between degrees at the end of each link and takes values between −1 and 1 [3]. A positive *r*-value indicates assortativity and a negative *r*-value indicates disassortativity. The assortative coefficient of this small network is −0.15, which suggests disassortativity. However, after removal of the 15 most connected nodes and their links, the assortative coefficient of the network becomes 0.28, which indicates an assortative correlation. Links for the 15 most connected nodes and the remainder of the network are shown in Figure 1C and D respectively, so that the topological similarity between them and the two schematic networks in Figure 1A and B can be noted. This phenomenon is not expected in a real disassortative network, where the network should remain disassortative when a few hubs are removed. One remarkable feature of the network in Figure 1D is the abundance of links between nodes with low connectivity, which can only be observed in an assortative network. Therefore, this protein network is likely to be a combination of disassortativity (Figure 1C) and assortativity (Figure 1D). This finding challenges the traditional opinion about biological networks being disassortative and requires a deeper investigation into their underlying degree correlation patterns.

### The dichotomy in correlation profile

**Figure 2:**
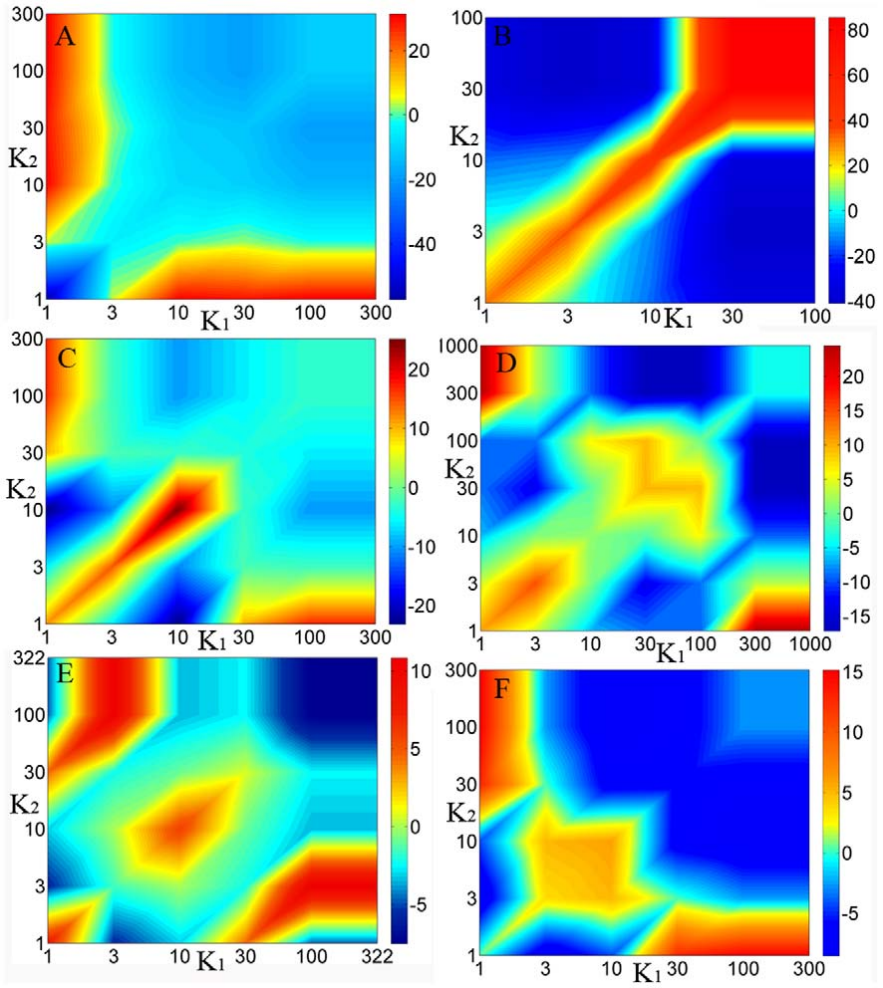
Correlation profiles of complex networks. **A.** The plot of Z-score of Internet at AS level which is known to be disassortative, where the red color reflects the affinity of nodes and blue color reflects the repulsion between nodes. **B.** The profile of a social network constructed by collaborations between authors who co-authored a paper, which is known to be assortative. **C.** The correlation profile of yeast PIN constructed by HC dataset. **D.** The correlation profile of yeast GIN. **E.** The profile of yeast metabolic network abstracted from KEGG. **F.** The correlation profile of HC dataset after removing interactions between proteins within the same complex. Note that through **C** to **F**, at least 99% nodes of biological networks are localized in the lower left corner where the diagonal is colored red.

The correlation profile provides the most direct hint to the current issue. A correlation profile compares the joint probability *P*(*K*_1_, *K*_2_) of finding a link between two nodes with degree *K*_1_ and *K*_2_ with the corresponding probability *P_r_*(*K*_1_, *K*_2_) in randomized networks [4]. Randomized networks are generated by random swapping of the links and thus preserve the degree distribution while having a neutral degree correlation. A plot of the value of Z-score, 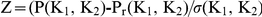, provides evidence of degree correlation that deviates from the uncorrelated neutral case as well as statistical significance [4]. Figure 2 shows the correlation profiles of the Internet at autonomous system (AS), known to be disassortative [10], an assortative social network [11], and three different types of biological networks, including physical interaction networks (PIN), genetic interaction networks (GIN) and metabolic networks.

For a real disassortative or assortative network, the color along the diagonal in the correlation profile is similar, reflecting the consistency of degree correlation pattern with which nodes of similar degree repulse (blue) or associate (red) with each other. This is seen in Figure 2A and B for the Internet and social network, known to be disassortative and assortative respectively. However, for biological networks, including PIN, GIN and metabolic networks, the color along the diagonal changes dramatically from red to blue, providing direct evidence for dichotomy in degree correlation (Figure 2C, 2D and 2E). In particular, a disassortative pattern is characterized by the blue region in the upper right corners and red regions in both the upper left and lower right corners, whereas an assortative correlation can be found in the lower left corner colored red. For example, there are 55 links between the top 1% most connected nodes in PIN, which is significantly less than the 123±10 links in randomized network. In contrast, there are 1,632 links between the top 10% most connected nodes (with the top 1% excluded), which is significantly larger than the 1,035±24 links in randomized networks. We further find the same dichotomy in the correlation profiles for many other datasets (Table 1 and Figure 1 in Text S1), suggesting that our finding is not influenced by the choice of datasets.

**Table 1:** Network datasets and their assortative coefficients *r*.

Dataset	Nodes	Links	*r*	*r* (number of hubs excluded)	Source
Internet at AS level	10670	22003	−0.186	−0.319 (100)	Leskovec et al, 2005
Collaboration network	5242	28980	0.659	0.672 (51)	Leskovec et al, 2005
HC dataset	4008	9857	−0.115	0.276 (40)	Batada et al, 2007
HC dataset (excluding protein complexes)	4008	6953	−0.137	0.094 (53)	Batada et al, 2007
FHC dataset	2559	5991	−0.064	0.234 (27)	Bertin et al, 2007
Ito dataset	3278	4549	−0.172	0.206 (49)	Ito et al, 2001
DIP dataset	5213	25232	−0.101	0.108 (97)	DIP (10/2010)
DIP-core dataset	2200	4514	−0.093	0.100 (17)	DIP (10/2010)
Genetic interaction network	3743	23125	−0.171	0.115 (83)	Biogrid(version 3.1.72)
Metabolic network(yeast)	1239	3611	−0.228	0.116 (20)	KEGG
Metabolic network(*E. coli*)	1208	3420	−0.196	0.146 (22)	KEGG
Metabolic network(*E. coli*)	765	2409	−0.177	0.205 (25)	Jeong et al, 2000
Metabolic network(*S. typhi*)	806	2539	−0.176	0.181 (24)	Jeong et al, 2000

In this study, the degree correlation of nodes in different biological networks shows the same dichotomy. That is, links between the most connected nodes are systematically suppressed, whereas those between nodes that are relatively loosely connected but have a similar degree are favored. In the correlation profiles in Figure 2, this suppression corresponds to the blue colored regions in the upper right corners of the diagonal, and the favored links corresponds to the red regions in the lower left corner of the diagonal. It is important to note that the correlation profiles use logarithmic coordinates, which means that only about 1% of nodes lie in the blue region in the upper right corners (note that 30≤k≤300 in the PIN correlation profile). Since hubs are usually defined as the top 10%–20% most connected nodes, it may be incorrect to conclude that “hubs” in biological networks are systematically suppressed [4], [12].

Although our results suggest an inherent consistency in topological organization of different biological networks, several other studies suggest that metabolic networks may have a different network topology [13]. Two reports show that hub nodes are more likely to be linked to each other in metabolic networks, whereas in protein networks the hubs are anti-correlated [14], [15]. Thus, these results seem to imply that metabolic networks have a fundamentally different network topology than other biological networks. However, other researches present contrary observations that links between hubs in metabolic networks are indeed suppressed [6], [8]. By comparing these results, we found that studies supporting the suppression between hubs use full datasets for metabolic networks, whereas studies supporting the affinity between hubs removed “popular metabolites” such as ADP, ATP and water, which correspond to the blue region in the upper right corners of our correlation profile (Figure 2E). In other words, the contradictory observations in the former studies arise from the dichotomy in degree correlation of metabolic networks. Therefore, our finding of dichotomy in degree correlation reconciles the contradictory observations for metabolic networks and again suggests that different biological networks may in fact have the fundamental network topology. The dichotomy in degree correlation may have possible applications, for example aiding the development of anti-cancer drugs. Hub genes of GIN are potential targets for anti-cancer drugs because cancer cells often carry a lot of cancerous mutation and thus may be destroyed preferentially by the inactivation of a hub gene which has synthetic lethal interactions with those mutations [16], [17]. The apparent dichotomy in degree correlation of GIN may suggest what kind of hub should be selected preferentially according to the degree distribution of cancerous mutations.

### Reproduced dichotomy in PINs excluding protein complexes

For PIN, the red region in the lower left corner of the correlation profile can probably be attributed to the fact that members of complexes tend to physically interact with other proteins from the same complex [4], [18]. To investigate this, we took a comprehensive catalogue of 408 manually curated yeast protein complexes reported in the current literatures [19], and reanalyzed the correlation profile of PIN excluding all interactions between proteins within the same complex. This removed about one third of the links from the original network. The average clustering coefficient of an HC network is above 0.16, while after this step it is below 0.06, suggesting that most of the densely connected regions have disappeared. Nevertheless, the dichotomy in the correlation profile of PIN remains (Figure 2E). The same analysis on another high-confidence dataset known as DIP-core shows the same result (see Figure 2 in Text S1) [20]. The physical protein networks, especially the high-confidence datasets, are believed to be enriched of protein complexes [21]. Thus, the reproduced dichotomy in the two high-confidence datasets further suggests that dichotomy is an inherent property of physical protein networks. Further evidence of dichotomy related to the affinity between low connected proteins (1≤k≤3) is unlikely to be attributable to multi-protein complexes.

Another source of evidence comes from the enhanced links between date hubs. It has been found previously that hub proteins of PINs can be partitioned into date and party hubs, and that most party hubs are members of protein complexes while most date hubs are not [22], [23]. A PIN constructed by a filtered high-confidence dataset (FHC) also shows the dichotomy in correlation profile (Table 1 and Figure 1 in Text S1), where a prominent red region along the diagonal can be found in the lower left corner, corresponding to proteins with degree less than 30. 236 party and 290 date hubs having degree less than 30 were identified according to the definition of the two types of hubs [24]. There are 465 links between these party hubs and 885 links between these date hubs in FHC dataset, both of which are significantly above 258±13 links and 539±17 links, respectively, in randomized networks (*P value*<0.01). The enhanced number of links between date hubs further suggests that the dichotomy can not be solely attributed to protein complexes. Thus the dichotomy in degree correlation is an inherent property of protein networks.

### The dichotomy determines neighborhood connectivity

**Figure 3:**
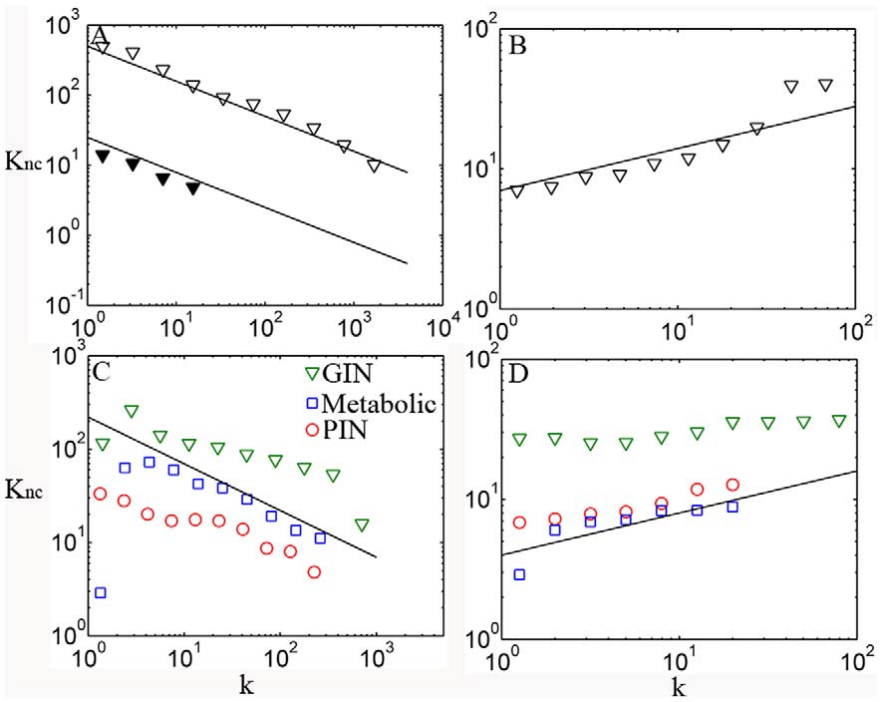
Correlations between node connectivity and its neighborhood connectivity. **A**. The nearest neighbors' average connectivity *K_nc_*, of nodes with connectivity *k* for Internet at AS level, and for the rest of network with top 1% best connected nodes removed (solid triangle). **B**. The same as **A** but for social network of co-authored relationship. **C**. Correlations of biological networks: PIN of HC dataset (red), GIN (green) and yeast metabolic network (blue). **D**. Correlations of biological networks after removing top ∼1% best connected nodes (detailed numbers of hubs removed are shown in Table 1). The solid lines in **A** and **C** correspond to 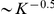; the solid lines in **B** and **D** correspond to 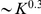. Note that the solid lines in **C** and **D** are not fitted to biological networks; they are drawn to compare with Internet and social network.

The degree correlation can also be extracted by studying the relationship between nodes' connectivity *k* and the average connectivity, *K_nc_*, of the nearest neighbors [4], [13], [25]. In Figure 3, we show the results of this for the above networks. For the Internet, *K_nc_* is consistent with a previous study in showing a clear power-law dependence, *K_nc_*∼*k^γ^*, with γ≈−0.5 (Figure 3A) [25]. For the social network, however, *K_nc_* shows a gradual increase with *k* (Figure 3B), suggesting a different pattern than for a disassortative network such as Internet. At first glance, the biological networks exhibit the same correlation pattern as Internet when considering the dependence of *K_nc_* on connectivity (Figure 3C). However, a huge difference emerges when the most connected hubs are excluded from analysis. The *K_nc_* for Internet decreases in the same rate after excluding the top 1% most connected nodes (Figure 3A), whereas *K_nc_* for the biological networks increases after excluding the most connected nodes, suggestive of an assortative correlation (Figure 3D). This observation gives additional credence to the dichotomy in degree correlation pattern of biological networks.

### The dichotomy of degree correlation determines a sickle-shaped distribution of clustering coefficient

**Figure 4:**
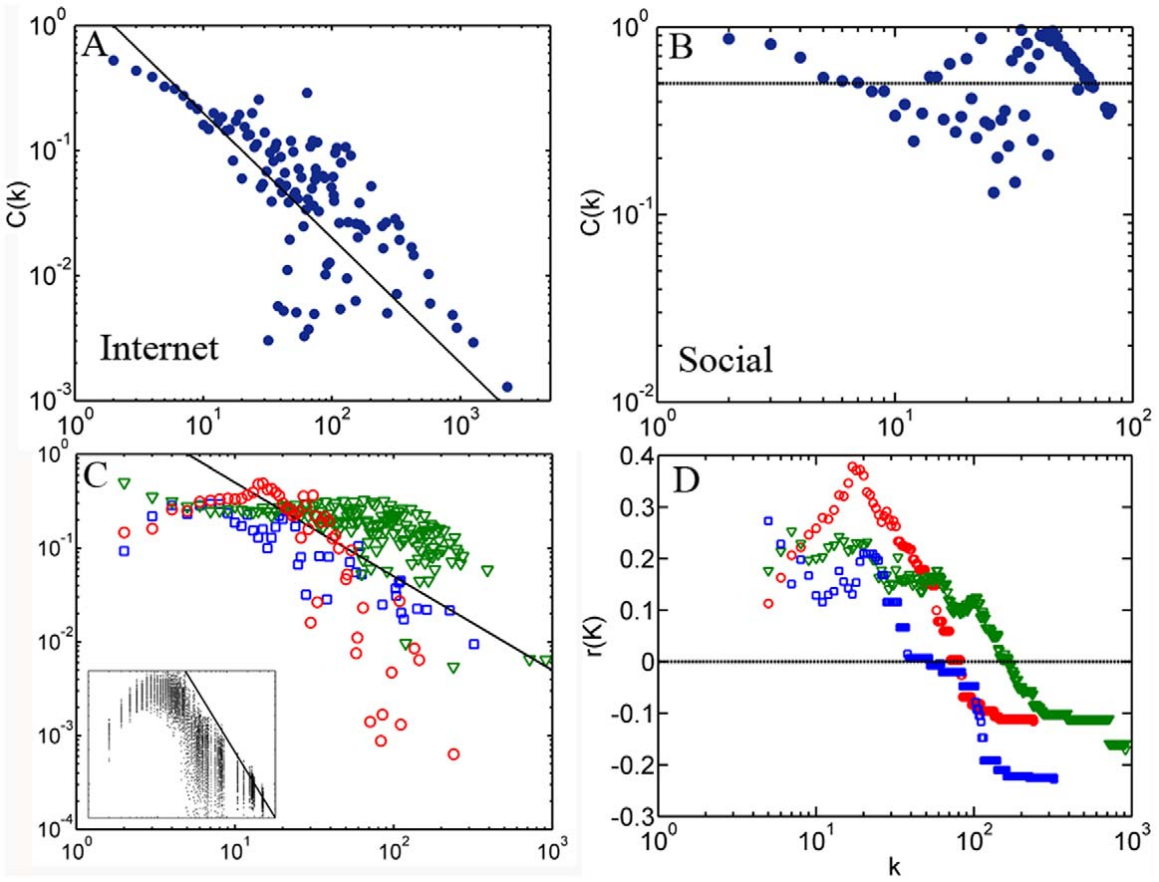
The dichotomy of degree correlation and its reflection on clustering coefficient distribution. The clustering coefficient distribution of Internet at AS level (**A**) and social network of co-authored relationship (**B**). **C**. The sickle-shaped *C(k)* curve of biological networks: PIN of HC dataset (red), GIN (green) and yeast metabolic network (blue). The inset displays the *C(k)* curves of 100 random dichotomized networks (each containing 10,000 nodes with *P*(*k*)∼*k* ^−2.4^, of which links of the top 0.5% best connected nodes are disassortative while those of other nodes are assortative). **D**. The assortative coefficient curves *r*(*k*) for the three biological networks. In **A**, **C** and **D**, the solid lines correspond to *C*(*k*)∼*k* ^−1^, which are drawn to compare with the hierarchical model.

Clustering coefficient denotes the proportion of links between the nearest neighbors of nodes [26]. In disassortative networks, highly connected nodes tend to be linked to low connected nodes (for example, the Internet, Figure 2A). As a result, a unique feature of disassortative networks not shared by either assortative or randomized networks is the gradual decline in the clustering coefficient with connectivity *k*. The clustering coefficient distribution, *C(k)*, of Internet at AS level is a perfect illustration of this theoretical speculation (Figure 4A). As a comparison, *C(k)* for the social network is relatively high for well connected nodes and does not decrease with connectivity (Figure 4B). For biological networks, however, we found a special form of *C(k)* that is distinct from the social network or Internet (Figure 4C). The value of *C(k)* for biological networks is relatively high at first and then suddenly decreases once *k* becomes large enough, which gives rise to a sickle-shaped distribution under logarithmic coordinates. This contrasts with the shape derived from a real disassortative network such as Internet. We also found the same sickle-shaped distribution for all the biological datasets shown in Table 1 (See Figure 3 in Text S1), all of which are consistent with the dichotomy in their correlation profiles.

To investigate whether this sickle-shaped distribution in *C(k)* reflects the dichotomy of degree correlation, we measured the *C(k)* distribution for 100 random dichotomized networks with the same degree distribution as biological networks (see materials and methods section for how to generate dichotomized networks). As shown in the inset of Figure 4D, similar to biological networks, the *C(k)* distribution for random dichotomized networks is also sickle-shaped. Ravasz et al. measured the *C(k)* distribution of metabolic networks for 43 organisms, and each of them shows a sickle-shape [26]. They proposed a hierarchical model to explain the *C*(*k*)∼*k* ^−1^ dependence at the tail of the distribution. However, the model dose not explain the sickle-shape of the entire *C(k)* distribution and dose not take into account the degree correlation pattern of networks. Another former study also suggests that the variations in the clustering coefficient with node degrees are mainly determined by the degree correlations [27].

We note that the assortative coefficient *r* does not distinguish between dichotomized and disassortative networks, but its variance *r(k)* is more discriminating when nodes with connectivity larger than *k* are excluded. Note that to reflect the degree correlation of the original networks, we do not remove the links incident with these notes from network and recalculate the degree for the rest of nodes. The distribution of *r(k)*, which varies with *k*, captures the dichotomy of degree correlation (Figure 4D). In particular, *r(k)* for biological networks starts from a high positive value and drops to below zero, indicating a switch in their degree correlation patterns from assortativity to disassortativity (Figure 4D). On the other hand, *r*(*k*) for Internet or social network never varies above or below zero (See Figure 4 in Text S1). One can find a connection by comparing distributions of *r*(*k*) and *C(k)*. *C(k)* decreases when there is a significant decline in *r*(*k*) (Figure 4C vs. D). A quantified connection can be measured using Pearson coefficients between *r*(*k*) and *C(k)*. The Pearson coefficients for PIN of HC dataset, GIN and yeast metabolic network are 0.78, 0.75 and 0.75 , respectively. The strong correlation between *r*(*k*) and *C(k)* suggests that the degree correlation does indeed determine the variation of clustering coefficient.

### Origin of dichotomy in degree correlation

Spatially isolated functional modules are considered to be fundamental building blocks of cellular organization [26], but the relationship between their presence and degree correlation in biological networks has not been systematically analyzed. We next demonstrate that a network comprised of functional modules necessitates the dichotomy in degree correlation, and that in turn, the dichotomy determines the modularity structure of a network to a great extent. A distinguished feature of modular networks is the existence of highly separated but densely interconnected modules, characterized by high local clustering. High clustering means that two neighbors of a node are more likely to be connected, resulting in a large number of triangles. Thus, the modular networks differ from random networks in two main respects: separated parts and many triangles within each part. In a disassortative network, however, hubs preferentially link to low connected nodes and vice versa, while the links between two hubs or between two low connected nodes are highly suppressed. As a result, local clustering in disassortative network is extremely small. For instance, it was reported that the number of triangles in Internet is significantly smaller than their randomized counterparts [28]. This property stands in sharp contrast with modular networks such as PIN, in which nodes are much more densely clustered than in random networks. For example, the PIN for the HC dataset has 8851 triangles, which is significantly greater than the 714±20 triangles in randomized networks with the same degree distribution. There are ways to increase clustering, for instance by fully connecting hubs as in assortative networks, but this arrangement also rejects modularity by ruling out the existence of separated parts within a networks.

**Figure 5:**
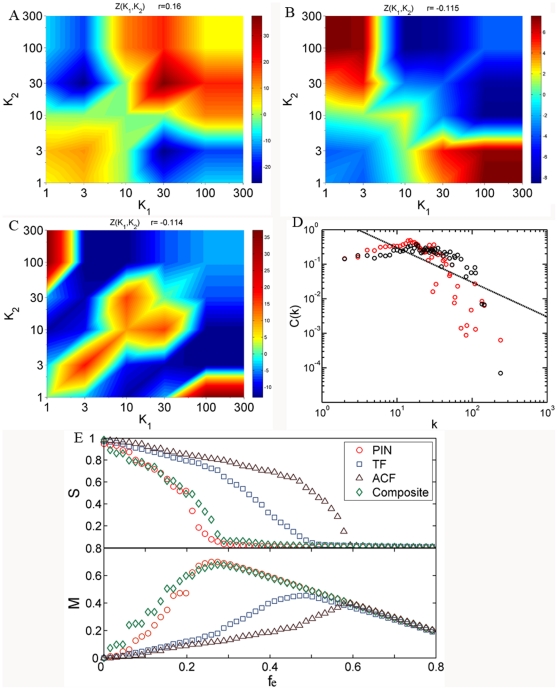
Degree correlation and modularity. **A**. The correlation profile of the triangle-favoring network. Note that the network is assortative. **B**. The correlation profile of the anti-correlation favoring network. Note that the network is disassortative. **C**. The correlation profile of the composite network, which presents a dichotomy in degree correlation. **D**. The two *C(k)* curves of the composite network (*T* = 1, black circles) and PIN of HC dataset (red circles) overlaps in a great extent. **E**. The strength of modularity, *M* and the relative size of largest component, *S* during the removal of a fraction *f*_e_ of intermodular edges for the triangle-favoring (TF) network, the anti-correlation favoring (ACF) network, the composite network and PIN of HC dataset.

Thus, the dilemma of modularity versus both disassortativity and assortativity exists, and this may require dichotomy in degree correlation. To prove this, we compare two different random networks with the same degree distribution as the PIN for the HC dataset. Both were generated by combining the edge rewiring step and Metropolis algorithm based on an energy function favoring the properties observed in PINs. To check whether a random network can evolve to a network exhibiting the properties observed in PINs, both networks start from a randomized version of the PIN that has 702 triangles. The first network favors the same number of triangles of PIN (N^Δ^ = 8851), which we refer to as a ‘triangle-favoring’ network. In this case the energy function is defined as 
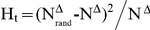
 and the network is sampled at a finite temperature 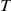. Rewiring steps that lower the energy function or leave it unchanged are always accepted, while those increasing it by ΔH_t_ are accepted with probability exp(−ΔH_t_/*T*). The triangle-favoring network generated by this method has 8683 triangles and assortative coefficient *r* = 0.16 (*T* = 0.1). From the red area around the diagonal of its correlation profile (Figure 5A), we can conclude that the large number of triangles favors assortativity as opposed to disassortativity. For the second network our algorithm was designed to generate the same level of anti-correlation as in the PIN, measured by the assortative coefficient *r*, and is referred to as an ‘anti-correlation favoring’ network. In practice, the *r*-value for a network with given degree distribution is solely determined by 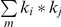, where 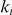 and 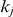 are the degrees of the nodes at the ends of the *m*th edge, with *m* = 1…9857. Thus, the energy function is defined as 
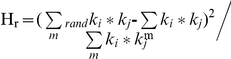
, and rewiring steps that lead to an increase of 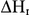 are accepted with probability exp(−ΔH_r_/*T*). The generated anti-correlation favoring network has the same level of anti-correlation as the PIN (*r* = −0.115) but has only 337 triangles (*T* = 10). Its correlation profile is shown in Figure 5B, and the blue color around the diagonal corresponds to disassortativity rather than dichotomy or assortativity. This result indicates that the level of anti-correlation favors links between nodes with different connectivities and preserves only a very small number of triangles. Therefore, the properties of high clustering and anti-correlation in biological networks are unlikely to present simultaneously in either a disassortative or an assortative network.

However, it is still uncertain that the dilemma necessitates the dichotomy in degree correlation observed in biological networks. This is easily checked by annealing a random network using a composite energy function that favors both the number of triangles and the level of anti-correlation of the PIN, which in our case can be defined as 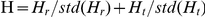, where the standard error here is evaluated by jackknife method (randomly removing an edge from the network each time) and is used to bring the two energy functions into the same order of magnitude. Rewiring steps are accepted with probability exp(−ΔH/*T*). We refer to the network generated by this composite energy function as a ‘composite’ network. Figure 5C shows the correlation profile of a composite network that has 8747 triangles and *r* = −0.114 (*T* = 1). It is apparent that the correlation profile is dichotomized in a way that is very similar to biological networks (Figure 2). Therefore, with regard to the dilemma we discussed above, the network does indeed evolve a dichotomy in degree correlation. We also plot the distribution of clustering coefficient of the composite network, which perfectly overlaps with the distribution of the real PIN (Figure 5D). This confirms our speculation that dichotomy in degree correlation determines a sickle-shaped clustering coefficient distribution.

We next show that, conversely, the dichotomy in degree correlation determines the strength of modularity in a network. A standard approach for quantifying the strength of modularity landscape in a network is to measure the number of intra-module links and compare it with what one would expect from chance alone [29], [30], [31]. For a given partition of network nodes into modules, the modularity of this partition is defined as:
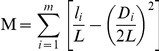
where *m* is the number of modules, *L* is the total number of links in the network, 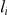 is the number of links within module *i*, and *D_i_* is the sum of degrees of all the nodes in module *i*. This definition implies that the value of *M* ranges from 0 to 1. Most real networks were found to have modularity between 0.3 and 0.7, while higher values are rare [30]. The value of *M* is high for a correct partition of a network that is modularly organized. An effective algorithm for discovering modularity structure involves iterative removal of edges from the network to divide it into modules, the edges removed at each step being identified by the largest betweenness value [32]. The iterative removal gradually separates modules from one another, and the inherent strength of the modularity of a network can be evaluated during these steps. Usually, the height of a peak of *M* is a good measure of the strength of the best modularity partition [30]. This algorithm was performed on the three random networks and the PIN of HC dataset, with the strength of modularity evaluated after removing a fraction *f_e_* of edges (Figure 5E). As we can see, although all the networks reach a peak at a finite fraction *f_e_*, the composite network and the PIN have a significantly stronger modularity compared with the triangle-favoring network or the anti-correlation favoring network. This is shown by the larger peak value of *M* and the smaller *f_e_* required to reach the peak. The peak value of PIN and the composite network are around 0.7, which usually indicates a strong effective modularity structure. It is also important to note that although PIN and the composite network share only 129 edges, their variances in *M* during the algorithm is almost the same, suggesting that the composite network has a similar modular organization as the PIN. We also plot the relative size of the largest component, *S*, as a function of *f_e_* (Figure 5E). Since modules are gradually separated from the network during the removal of intermodular edges, *S* should also gradually decrease and the rate of decrease should be similar for networks with similar modular organization. Thus, the variance of *S*, again, indicates that the composite network has a similar modularity structure as the PIN. We also show the four networks with the top 3,000 intermodule edges been removed (See Figure 5 in Text S1), from which we see that the PIN and composite network indeed have similar modules whereas the triangle-favoring network and the anti-correlation favoring network obviously lack modularity structure. Together, these results imply that the dichotomy in degree correlation is the topological foundation of the modularity structure in biological networks.

### Robustness and interconnectivity in the dichotomy of degree correlation

Discussing the influence of degree correlation on robustness and interconnectivity of networks is of fundamental importance as it implies an evolutionary advantage behind its existence. Disassortative networks, such as the Internet, are known to be extremely vulnerable to intentional attacks on hubs [6]. In disassortative networks, hubs connect to a large number of low connected nodes (as in Figure 1A and 1C), so that removing every single hub results in many isolated nodes, and even attacking only a few of hubs attacks all parts of the network at once. On the other hand, in assortative networks, hubs are fully connected and so attacking them is somewhat redundant [6]. However, this arrangement does not preserve the interconnectivity as well, since the affinity between low connected nodes reduces the possibility that they are connected to the largest component of the network and enlarges their distances to other nodes in the network. We next show that the dichotomy of degree correlation significantly increases the robustness while preserving the interconnectivity of a network.

**Figure 6:**
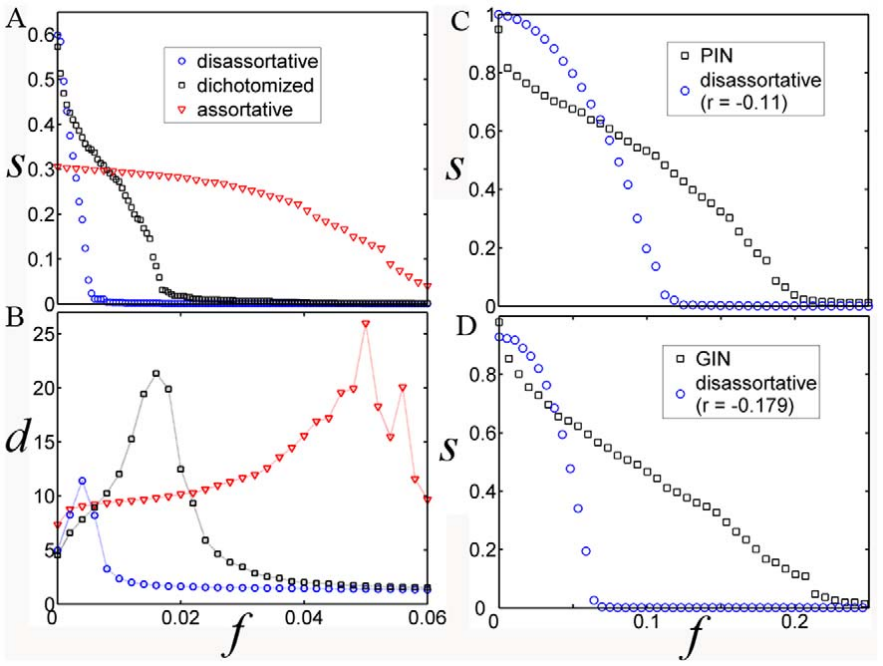
Robustness and interconnectivity of the network under targeted attacks. **A**. Comparison of the size of the largest component between disassortative, assortative and dichotomized network models, each containing 10,000 nodes with *P*(*k*)∼*k* ^−2.4^. **B**. Comparison of network diameter as a function of the fraction *f* of removed best connected nodes. Note that the size of the largest component of unperturbed dichotomized network (*f* = 0) is larger than that of assortative network, while the diameter is smaller than it, indicating that the dichotomy of degree correlation generates a more interconnected web. **C**. The size of the largest component in function of *f*, between PIN of HC dataset and a random network with similar level of anti-correlation (generated by rewiring links of PIN and by setting the parameter *p* = 0.05, see materials and methods). **D**. The same as **C** but between GIN and a random network with similar level of anti-correlation (generated by setting the parameter *p* = 0.1).

The analysis is carried out on random assortative, disassortative and dichotomized networks with the same number of nodes (N = 10,000), the same degree distribution that approximates to biological networks (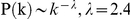), and thus the same number of edges (see materials and methods section for the construction of random networks). The difference between these networks is solely attributed to degree correlation pattern. The average assortative coefficient *r* of 100 assortative networks is 0.129 (±0.0001) and *r* of disassortative networks is −0.088 (±0.0005). For dichotomized networks, only the 0.5% most connected nodes are disassortatively linked to other nodes, whereas the rest of the nodes are assortatively linked, giving rise to a dichotomy in degree correlation with the average *r* being −0.079 (±0.003) or 0.14 (±0.01) after excluding the 0.5% most connected nodes. Thus, even 99.5% of nodes in a dichotomized networks are assortatively connected, the assortative coefficients is probably below zero. This fact might be mistaken as an evidence of disassortativity, as has previously been concluded for biological networks. Figure 6A and 6B show a comparison between the three types of network. The two figures plot the size of the largest component in each network, *S* and network diameter, *d*, as a function of the fraction of nodes removed [33].

The assortative network is certainly the most robust against attacks on nodes, which is indicated by both the slow decrease in *S* and the slow increase in *d*. However, it is also the worst from the perspective of interconnectivity, shown by the large diameter and the small size of *S* for the original network (*f* = 0). On the other hand, the disassortative network shows better interconnectivity but poor robustness against removal of nodes compared with the assortative network. For the dichotomized network, a significantly larger number of nodes must be removed to destroy the network than for the disassortative network (shown by *S* tending to 0 and the peak in *d*), and is surprisingly well connected compared with the disassortative network (large *S* and small *d* at *f* = 0). This result indicates that the dichotomized network takes full advantage of the links available to it, giving rise to the simultaneous appearance of robustness and interconnectivity within a single network. A comparison between two biological networks (see PIN of the HC dataset and the yeast GIN) and their disassortative counterparts with the same size and similar level of anti-correlation is also shown (Figure 6C and 6D), with both biological networks showing similar interconnectivity but significantly higher robustness than disassortative networks.

Two recent studies revealed that only about 0.5% nodes are truly critical for interconnectivity of protein networks [34], [35]. Our work provides a reasonable explanation for this finding. Although we do not compare the resilience of networks under random failures, considering that 99.5% of nodes in dichotomized networks are assortatively connected, it is reasonable to expect a case similar to targeted attacks on hubs. Supposing that interconnectivity and robustness are two parameters both relevant to evolution, this result suggests an evolutionary motivation for the existence of dichotomy in degree correlation of biological networks.

## Discussion

### A heuristic model of modular organization

**Figure 7:**
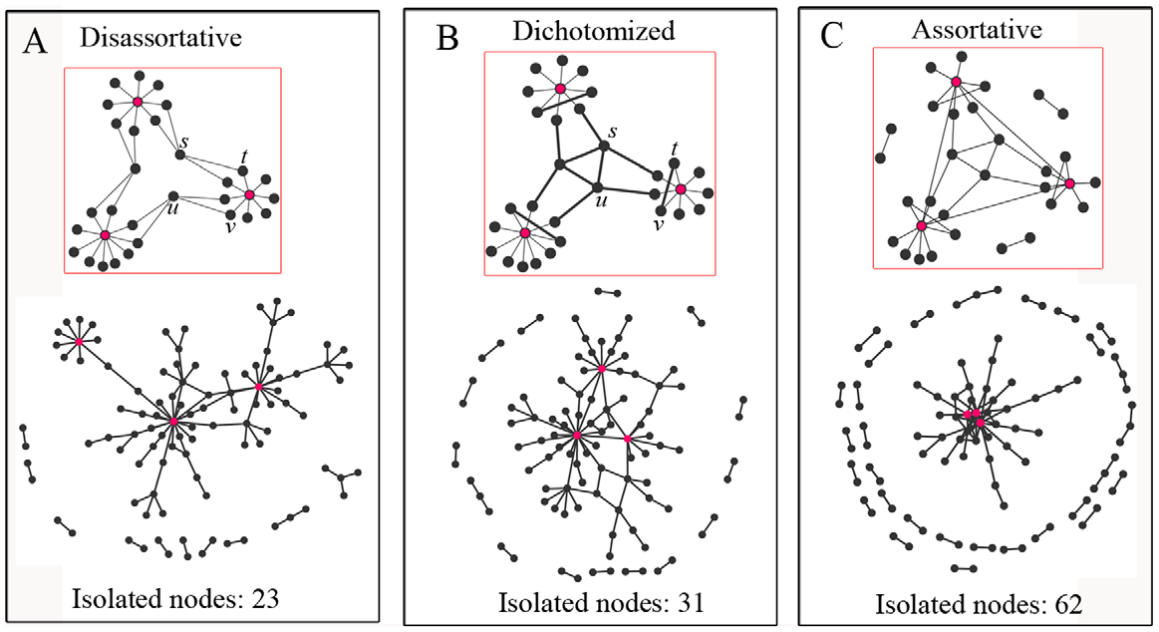
Schematic models of disassortative, dichotomized and assortative networks. **A**. Schematic model of a disassortative network (inset) and a random network under the principle of the model. **B**. The same as **A** but for dichotomized network. **C**. The same as **A** but for assortative network. Each of the random networks has the same number of nodes (100), the same number of edges (94) and the same degree distribution. Disassortative network model in **A** suggests a modular structure with modules separately distributed and pair-wisely connected, whereas **C** suggests a highly integrated network with nodes integrated by a core of fully connected hubs. The dichotomized network in **B** suggests a centrally connected modular structure where modules are tightly connected to each other rather than dispersedly distributed.

A complete understanding of the modularity structure in a biological network also depends on uncovering the organized principle according to which the network connects modules together [22]. Although our results indicated that the dichotomy in degree correlation is necessary for the existence of modules, it still raises the question of connection between different modules. The anti-correlation between the most connected nodes decentralizes the network and suggests nodes organized around dispersed hubs (Figure 7A), whereas affinity between hubs suggests nodes integrated by a fully connected central core (Figure 7C). Thus, the dichotomy in degree correlation may suggest modules that have been separated by the repulsion between a few well connected hubs, but that are centrally connected by a core formed by another group of relatively low connected hubs. This can be illustrated by a heuristic model of modular organization, which we refer to as “centrally organized modularity” (see the inset of Figure 7B). In this model, links for the three most connected hubs (red nodes) are disassortative, as represented by the suppressed links among them and the enhanced links between them and low connected nodes. On the other hand, the remainder of the network is assortative (shown by bold edges). The architecture of such a network integrates assortativity and disassortativity, and thus is dichotomized. Its apparent feature of centrally organized modularity cannot emerge from either a disassortative network model (inset of Figure 7A) or an assortative network model (inset of Figure 7B). Random rewiring steps would inevitably lead to a centrally connected modularity structure. For example, to generate a random dichotomized version of network, we use the disassortative network as a seed to rewire edges. Two randomly selected edges (i.e., (s, t) and (u, v)) that are not connected to any of the three most connected nodes are replaced by two new edges that are assortatively connected (i.e., replaced by (s, u) and (t, v)). Thus, repeated random rewiring steps also result in a centrally connected network, as shown in the inset of Figure 7B.

To further investigate the modular organization, we plot three small random networks using the principle of these models (Figure 7 A–C). Each network has the same number of nodes (N = 100), and the same degree distribution (generated according to 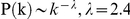, See Materials and Methods). Thus the difference in the organization solely attributes to the different degree correlation patterns. For the network of disassortative model, although modularity is visible through connecting nodes with the dispersed hubs, these modules are spread too far apart from each other. Conversely, for the network of assortative model, although it is highly integrated because of the affinity between hubs, the modularity structure does not preserved and too many nodes are isolated from the largest component. The network of the dichotomized model, however, preserves both the modularity and integration of the network (Figure 7B).

### Biological significance of the dichotomy

**Figure 8:**
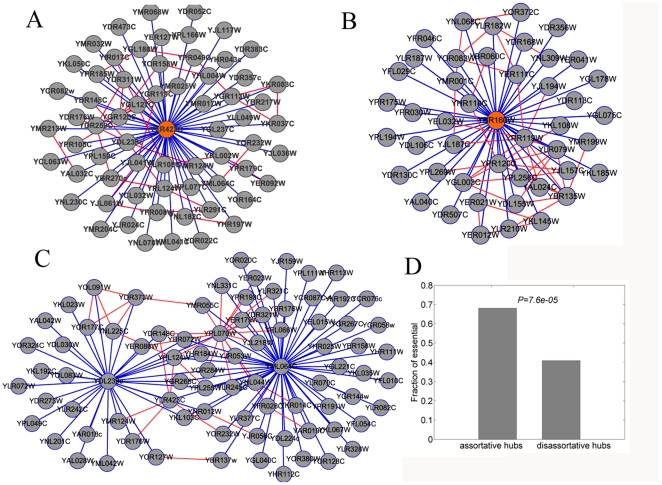
Dichotomous modules. **A**. Modules organized around YLR423C YBR160W. Assortative links are colored red and disassortative links are colored blue. **B**. Modules organized around YBR160W. **C**. Two modules organized around YDL239C and YML264C are connected to each other through assortative links. D. Assortative hubs are more essential than disassortative hubs (chi-square test).

Figure 8A–B shows the subnets consisting of proteins organized around super-hubs YLR423C and YBR160W according to the HC dataset. The first subnet around YLR423C forms a module that is essential for autophagy, while the later forms a module that is essential for the start of cell cycle [36], [37]. Both the two subnets consist of a lot of interactions between the super-hub and low connected protein pairs (blue), as well as a lot of interactions between their neighbors that have similar degrees (red). In these two subnets, red and blue edges correspond to assortative links and disassortative links respectively. As we can see, many red links are necessary to ensure the high local clustering of super-hubs given the fact that super-hubs are preferentially connected to non super-hubs. On the other words, the only way to keep high density of inner modular links is the dichotomy of degree correlation. Biological networks show many modules just like these (see Figure 5 in Text S1). Figure 8C shows two modules organized around super-hubs YDL239C and YML264C in the HC dataset. YDL239C is a protein involved in the pathway that organizes the prospore membrane (PSM) during sporulation, and YML264C is a GTP-binding protein involved in termination of M phase. Both the two modules consist of many blue links and red links, indicating the dichotomy of degree correlation. Consistent with the strong repulsion between super-hubs, the two hub proteins do not interact directly. However, the two modules are tightly integrated by the red links, supporting our heuristic model of modular organization as illustrated in Figure 7C. Therefore, realizing that biological networks are dichotomous provides a new insight into the principle according to which the network modules are organized.

According to the dichotomous nature of biological networks, nodes can probably be divided into two classes by their roles in degree correlation. To further investigate the biological significance of the dichotomy, we divide hub proteins into two distinct classes and study their relationship with gene lethality (see Materials and Methods section). We obtain 204 assortative hubs and 66 disassortative hubs in total. The average degree of assortative hubs is 17.6, whereas that of disassortative hubs is 38.4. According to previous studies that gene lethality is positively correlated with the connectivity of that gene in biological networks, the disassortative hubs should be more essential than assortative hubs. However, our result yields a surprising finding. That is, the assortative hubs are significantly more essential than the disassortative hubs. A possible explanation for the high lethality of assortative hubs is that assortative hubs are responsible for inter-module connecting. Modules are otherwise poorly connected to each other, so the failure of assortative hubs will probably have a large impact. On the contrary, disassortative hubs are within modules so that the failures of them are confined to their module and thus have a relatively smaller impact. Furthermore, the anti-correlation between disassortative hubs and well connected proteins may suppress their propagation of deleterious perturbations over the network, so that the failures of them are not lethal. A previous study once found that the bottleneck genes are more essential than other genes [34]. More interestingly, a study on metabolic networks also found that module connectors are more important than intra-module hubs; even their connectivity is significantly smaller than intra-module hubs [38]. Therefore, the dichotomy of degree correlation leads to a better understanding of the knockout phenotypes.

It is believed that the degree correlation of protein network reduces the number of interactions between essential proteins (IBEPs), thus providing some kind of protection for cellular system [4], [39], [40]. Since we have shown that biological networks are dichotomous in degree correlation, there is a need to re-evaluate this concept. For this purpose, we calculate the number of IBEPs in random networks with no degree correlation and random networks with the same dichotomous degree correlation as PIN (see Figure 6 in text S1). We find that, by keeping the dichotomy of degree correlation, the number of IBEPs is significantly larger than the number of random networks without degree correlation. Nevertheless, both of them are significantly smaller than PIN. Thus, although the number of IBEPs is reduced in random networks, it is not because of degree correlation but because of other possible reasons, i.e., the existence of essential protein interactions [40]. We therefore suggest to reconsider the relationship between the role of IBEPs and topological structures of protein network [39].

In summary, while current studies widely believe that complex networks are either disassortative or assortative [41], we find a dichotomy in degree correlation of different biological networks. This finding distinguishes biological networks from two networks of different areas. We suggest that many topological measures and biological significance related to these topological measures should be re-evaluated under this new finding. It also suggests a novel model of modular organization, which resolves the conflict between the specificity of modules and the integrity of network. Hence, a network with dichotomy in degree correlation may better integrate information and resolve conflicts of different modules. This shows an intriguing similarity with neurobiology, where the central nervous system integrates information from different senses and resolves conflicts [42]. It is reasonable to suppose that some other networks believed to be disassortative and modular, such as WWW, are probably dichotomized too. Assortativity alone can also generate modularity structure, for example by fully connecting the same type of node while suppressing links between nodes of different types. This mechanism, however, in spite of its advantage in explaining the formation of community structure in social networks and the formation of small biological modules such as protein complexes, cannot generate the kind of large-scale modules observed in biological networks.

## Materials and Methods

### Datasets

The PIN of the HC dataset in the main text is constructed by a high-confidence dataset curated from the literature and high-throughput sources such as Y2H, which contains 9857 interactions between 4008 proteins after excluding redundant edges [9]. The GIN is constructed by 12,100 synthetic lethality genetic interactions and 15,322 synthetic growth defect interactions between 3743 proteins, downloaded from Biogrid database [43], with genes having no “Systematic Names” excluded. We analyze metabolic networks obtained from two different sources: KEGG and the datasets of Jeong et al. [44], [45]. The yeast metabolic network of KEGG is used in the Figure 2 and the others can be found in Text S1. Metabolic networks for *E. coli* and *S. typhi* from Jeong datasets display similar dichotomous patterns (See Figure 1 in Text S1 and Table 1). We also analyzed a number of other datasets [24], [43], [45], [46], for which the descriptions can be found in Table 1 and the correlation profiles can be found in Figure 1 of Text S1. The list of lethal yeast genes is downloaded from **(** http://www-sequence.stanford.edu/group/yeast_deletion_project **).**

### Random networks construction

For our purpose on comparing robustness and interconnectivity of networks with different degree correlation patterns and the same degree distribution, we use a simple method proposed by Newman to generate random networks [6]. Specifically, the method is as follows. First, we form a node set *O* containing *k_i_* copies of node *i* from any given distribution (We use 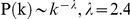 in our analysis, which approximates the PIN well). Then we connect nodes of *O* randomly in pairs to generate a neutral uncorrelated scale-free network. This step has also been described by Molloy and Reed [47]. One limitation of this step is the appearance of multiple edges connecting the same pair of nodes. To prohibit these multiple edges, we randomly swapping the edges in the network until no multiple edges between two nodes exit. Next, we choose at random two edges from the network generated by the above step, for example (a,b), (c,d). Then, we measure the remaining degrees (degree minus 1) of the nodes at the ends of the two selected edges, denoting these by 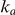, 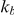, 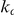, 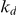. We now replace the two edges by two new edges (a,c), (b,d) with probability 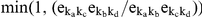, where *e_ij_* is the joint probability of nodes. In our analysis, *e_ij_* has the following follows a symmetric binomial form:
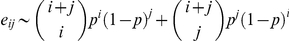
One can get assortative networks or disassortative networks by changing the parameter *p* in the formula above. In our analysis, *p* is set to 0.5 to generate an assortative network and 0.05 to generate a disassortative network. To generate dichotomized networks, we set *p* to 0.05 for the joint probability between the top 0.5% best connected nodes and 0.5 for the joint probability between the rest of nodes. This gives rise to a dichotomy in degree correlation with an *r*-value below 0 initially and above 0 after excluding the 0.5% most connected nodes. The networks generated have the same number of nodes, the same degree distribution, allowing a comparison of the difference between network properties solely attributed to degree correlation pattern. A more detailed description of this method can be found in the work of Newman [3], [6].

Neutral randomized networks used in the analysis of Figure 2 are generated in a slightly different way: We use Internet, social network and biological networks as seed networks, then select two edges at random and replace them by two new edges, as described by Malsov and Sneppen [4]. We then repeat the rewiring step until every edge in the network is rewired at an average of 100 times. 100 randomized networks are generated using the above procedure for each seed network to determine the standard deviation 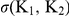 of Z-score.

To create random networks with the same degree correlation as biological networks, we propose the following algorithm.

(1) We estimate the joint probability *P*(k*_i_*, k*_j_*) of two nodes (with degree k*_i_*, k*_j_* at either end of a randomly chosen edge in biological network. The joint probability should satisfy the sum rules
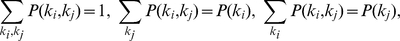



Where *P*(*k*) is the degree distribution of biological network and *P*(k*_i_*), *P*(k*_j_*) denotes the probability of a random chosen nodes with degree k*_i_* and k*_j_*. *P*(k*_i_*,k*_j_*) is the neighborhood degree distribution of node with degree k*_i_*.

(2) For each degree *k*, we draw *N*•*P*(*k*)nodes from the degree distribution *P*(*k*), and then form a node set S containing k*_i_* copies of each node *i*, where *N* denotes the number of nodes in biological network. Thus, the number of elements in *S* is the number of ends of edges in biological network.

(3) We select at random two nodes from *S*, connect them to generate a new random graph and then remove them from *S*. At each time, we estimate the probability *P_r_*(k*_i_*,k*_j_*) in the random graph 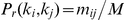, where *m_ij_* is the number of edges connecting nodes of degree k*_i_* and k*_j_*. Note that k*_i_* and k*_j_* are not re-estimated in the new graph; they are fixed attributes of nodes of S. So, 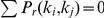 in the beginning. Then we test if 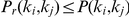, and when the condition is not fulfilled, we discard the two nodes and draw two new ones from *S*. We repeat this step until 
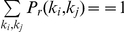
.

This algorithm was used to test the speculation that dichotomy in degree correlation determines a sickle-shaped clustering coefficient distribution (see Figure 7 in text S1).

### Hub partition

For each hub (defined as the 10% of most connected nodes), we calculate its neighbourhood degree distribution and compare it with what one would expect in random networks. We identified 204 hubs that have significantly more hub-hub connections, which we denoted by assortative hubs; 66 hubs that have significantly fewer hub-hub connections, which we denoted by disassortative hubs (Kolmogorov-Smirnov test, *p* = 0.05). In practise, one can use the formula 
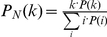
 to estimate the neighbourhood degree distribution in random networks.

## Supporting Information

Text S1 **Supporting information.**
(DOC)
