# Supplementary material for: The Dichotomy in Degree Correlation of Biological Networks
Source: PLoS One. 2011 Dec 2;6(12):e28322. doi: 10.1371/journal.pone.0028322 (PMC3229552; doi:10.1371/journal.pone.0028322)
Supplement: Text S1 — Supporting information. (DOC) [file pone.0028322.s001.doc]

**
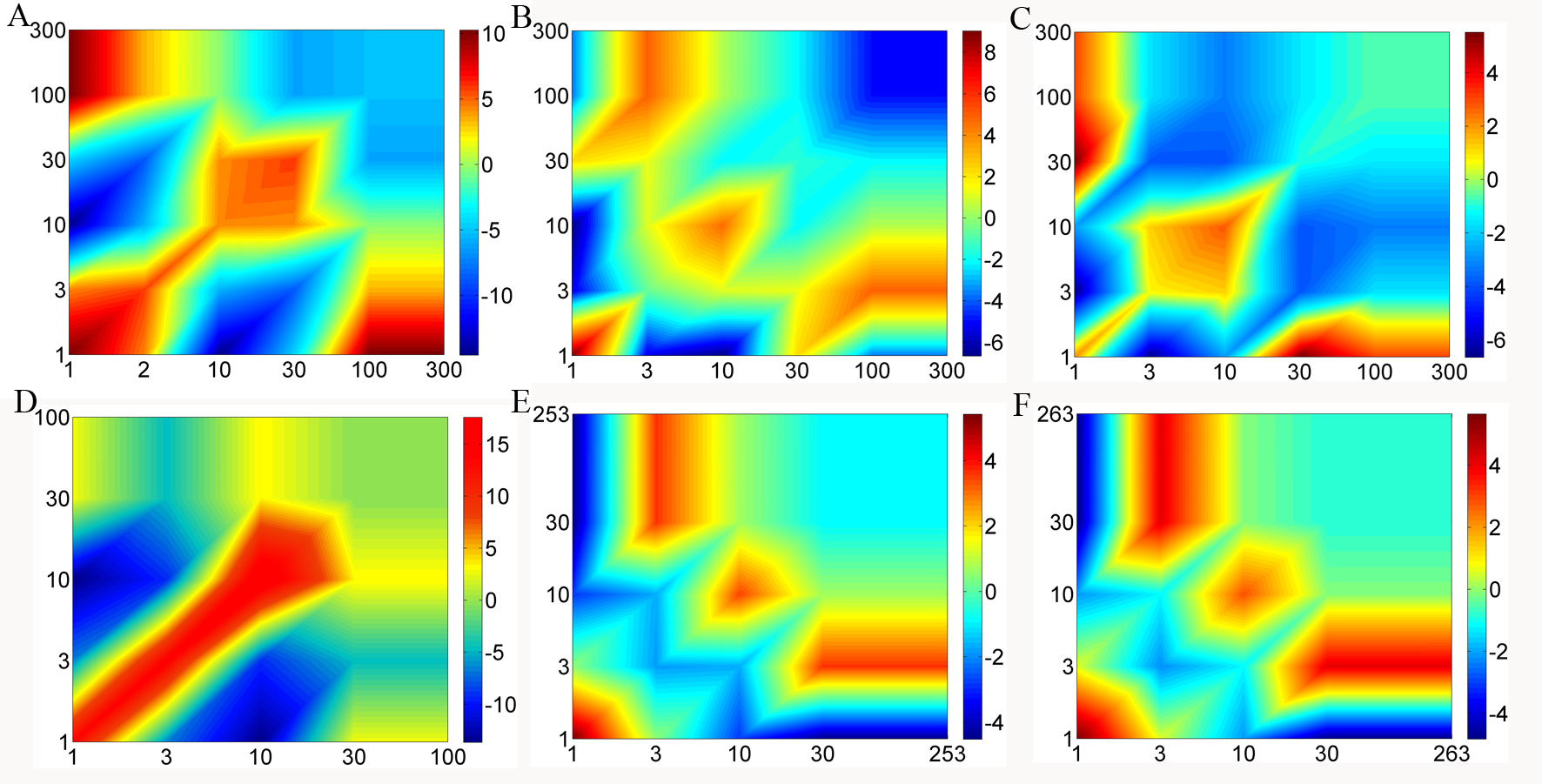
**

**Supplementary Figure 1.** The dichotomy in correlation profile of DIP (**A**), metabolic network of E.coli from KEGG (**B**), Y2H dataset of Ito (**C**), FHC dataset (**D**), metabolic network of E.coli (**E**) and S. typhi (**F**) from Jeong H et al datasets. A description of the datasets can be found in Table 1. The color through A to D is plotted by Z-score (), where the red color indicates the enhanced links between nodes compare to randomized networks, and blue color reflects the suppressed links between nodes.


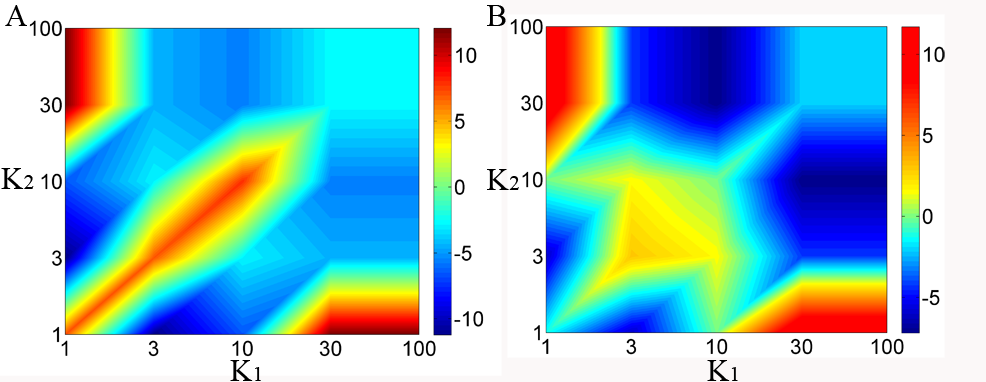


**Supplementary Figure 2.** The correlation profile of DIP-core dataset (**A**) and the same dataset after interactions between protein pairs within the same complex removed (**B**). The DIP-core dataset has 4514 interactions between 2200 proteins, of which 1529 interactions are detected to be between members of the same protein complex, according to 408 curated yeast protein complexes reported in current literatures. The physical protein networks, especially the high-confidence datasets, are enriched of protein complexes. Thus, the reproduced dichotomy in this high-confidence dataset further suggests that the dichotomy is an inherent property of physical protein network. It is important to note that by deleting all the links between protein pairs within the same complex, the connection between these proteins are actually underestimated.


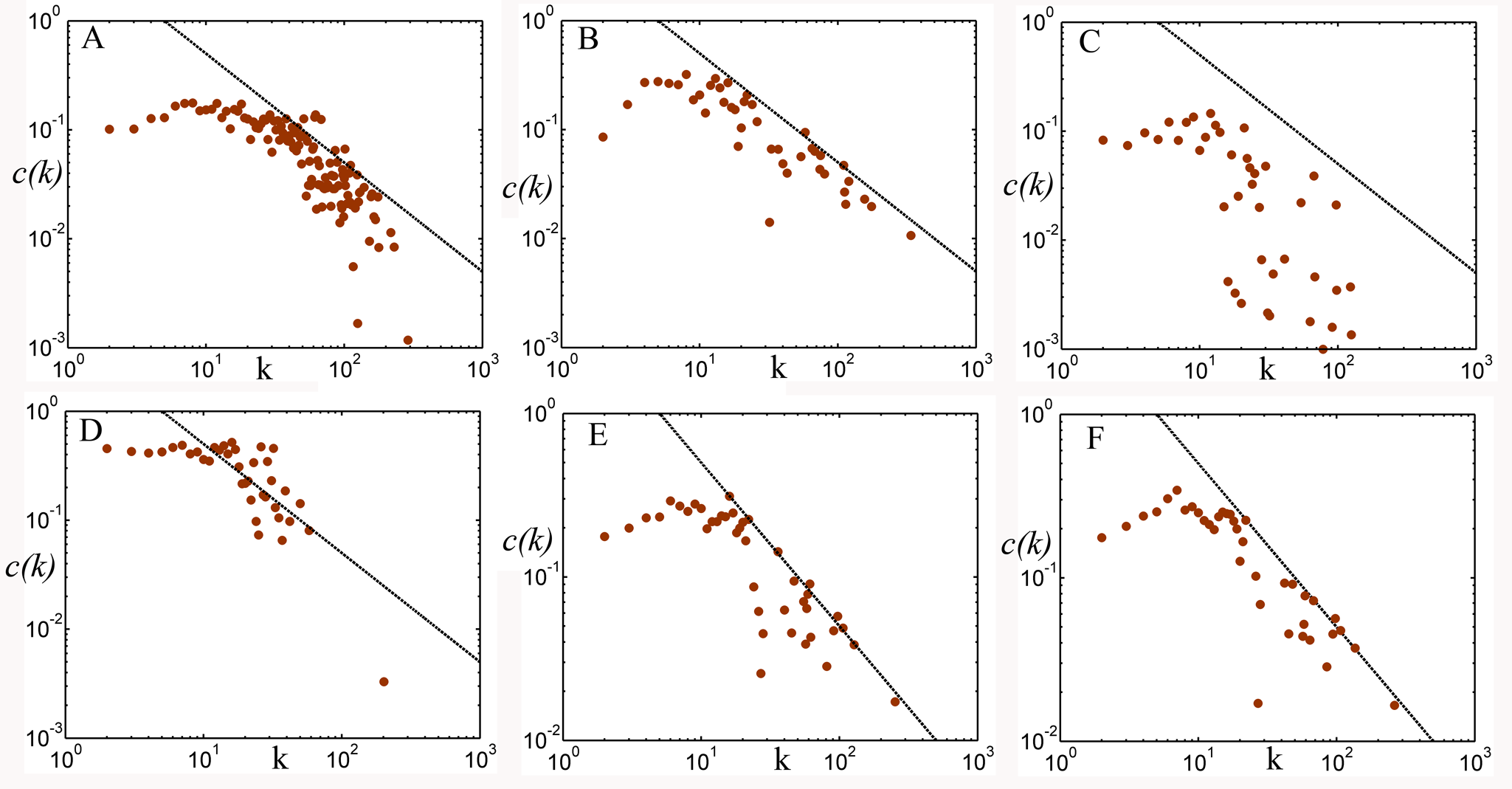


**Supplementary Figure 3.** The sickle-shaped clustering coefficient distribution of DIP (**A**), metabolic network of E.coli from KEGG (**B**), Y2H dataset of Ito (**C**), FHC dataset (**D**), metabolic network of E.coli (**E**) and S. typhi (**F**) from Jeong H et al datasets. The dashed lines through A to D correspond to $\text{C}\left(\text{k}\right)\sim k^{-1}$.


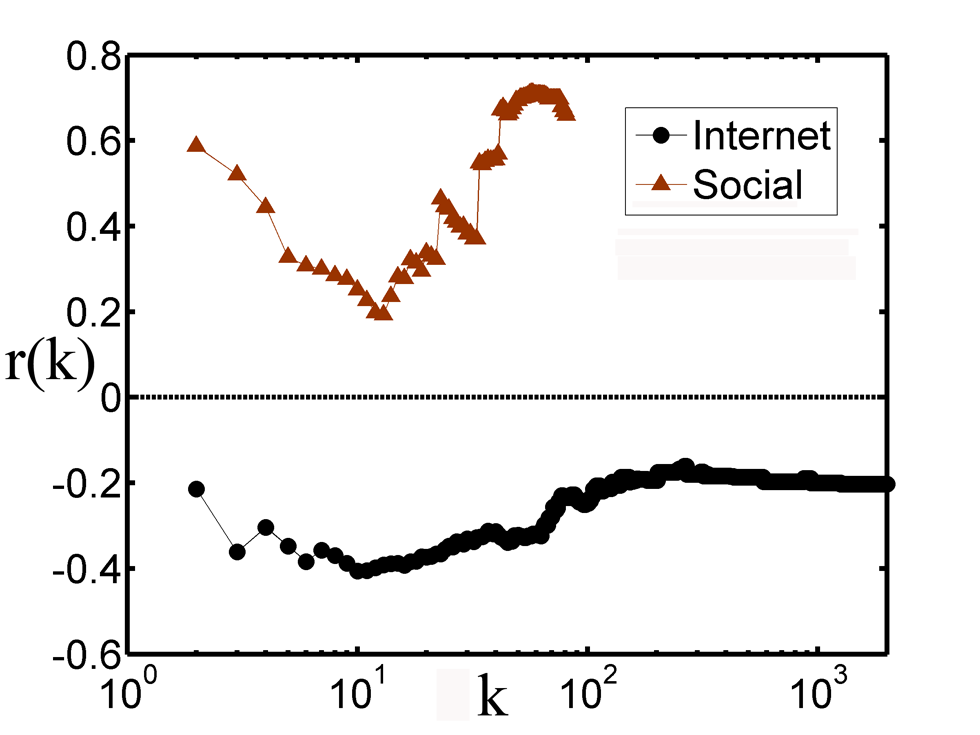


**Supplementary Figure 4.** The assortative coefficients $\text{r}\left(\text{k}\right)$ for Internet at AS level and for social network of co-authored collaborations, in function of *k* that represents nodes with degree larger than *k* excluded. The assortative coefficients of Internet and social network never vary above or below zero, in contrast to biological networks (see Figure 4).


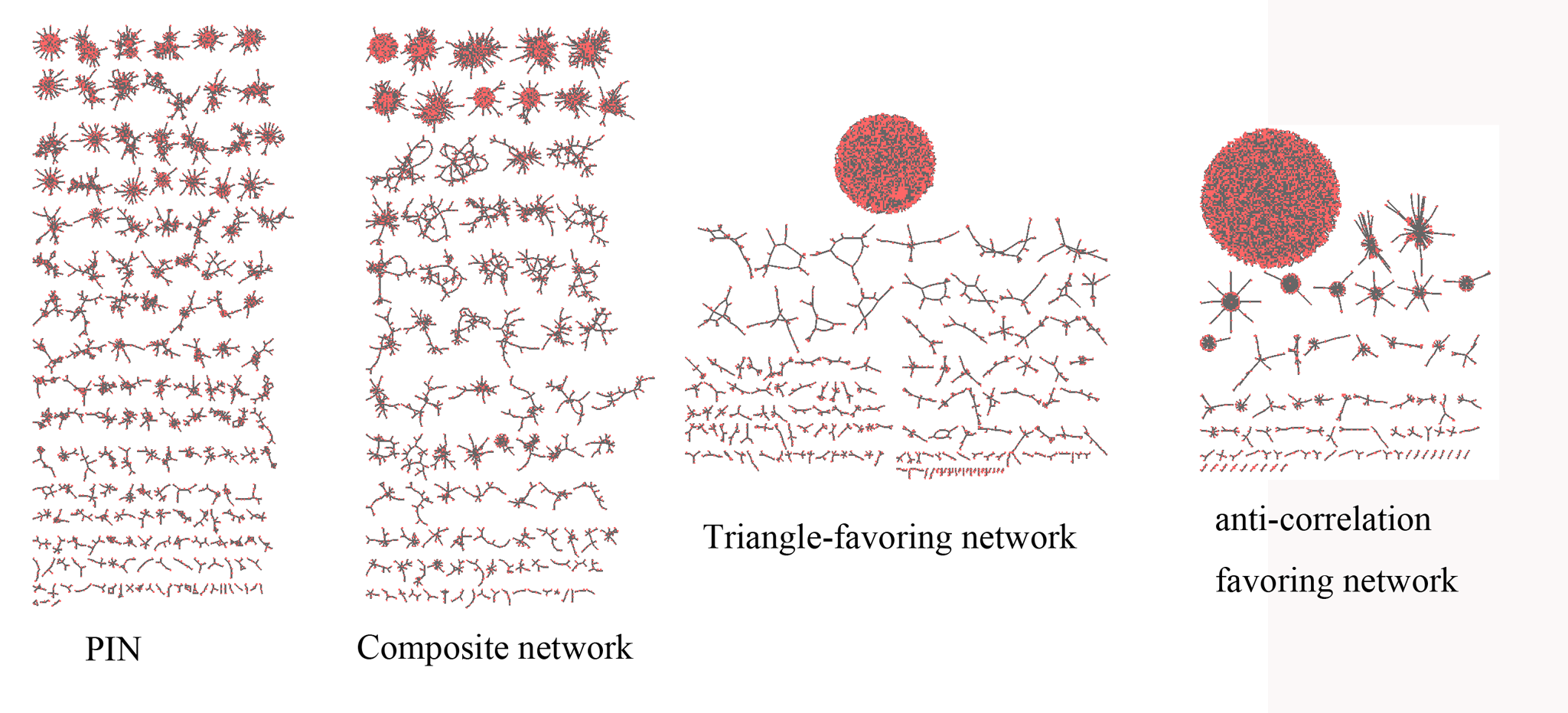


**Supplementary Figure 5.** The largest component of the four networks after removal of the top 3,000 edges identified by the shortest-path betweenness algorithm. One can see that PIN and the composite network have similar size modules whereas the triangle-favoring network and the anti-correlation favoring network obviously lack modularity structure. This result suggests that both the anti-correlation between hubs and the high clustering are necessary for the existence of modularity, which is probably the origin of dichotomy in degree correlation (see Figure 5C).


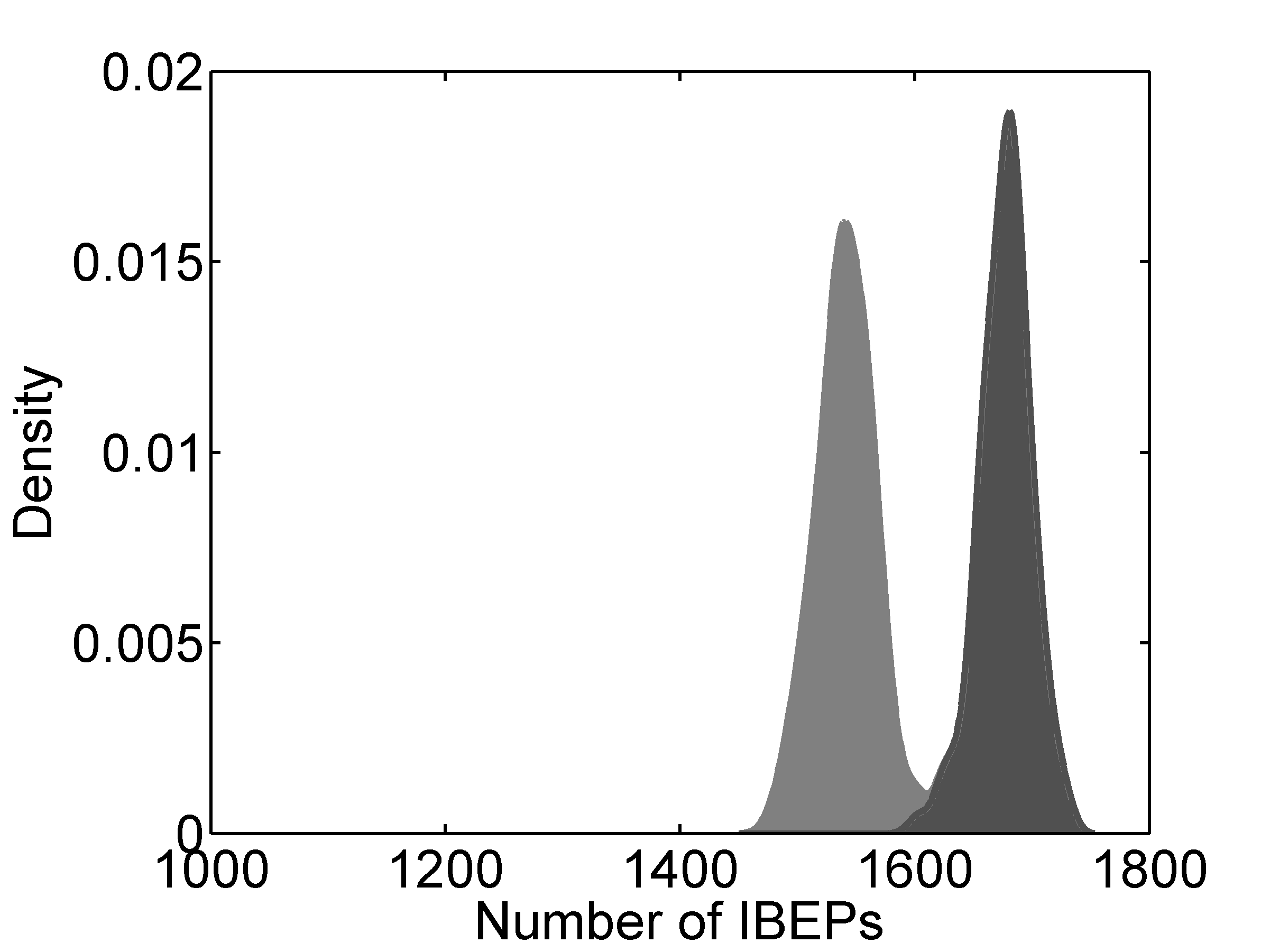


**Supplementary Figure 6.** By keeping the degree correlation, the number of IBEPs (Black) are significantly larger than the number of random networks without degree correlation (Gray). Nevertheless, both of them are significantly smaller than PIN, of which the number is 2273.


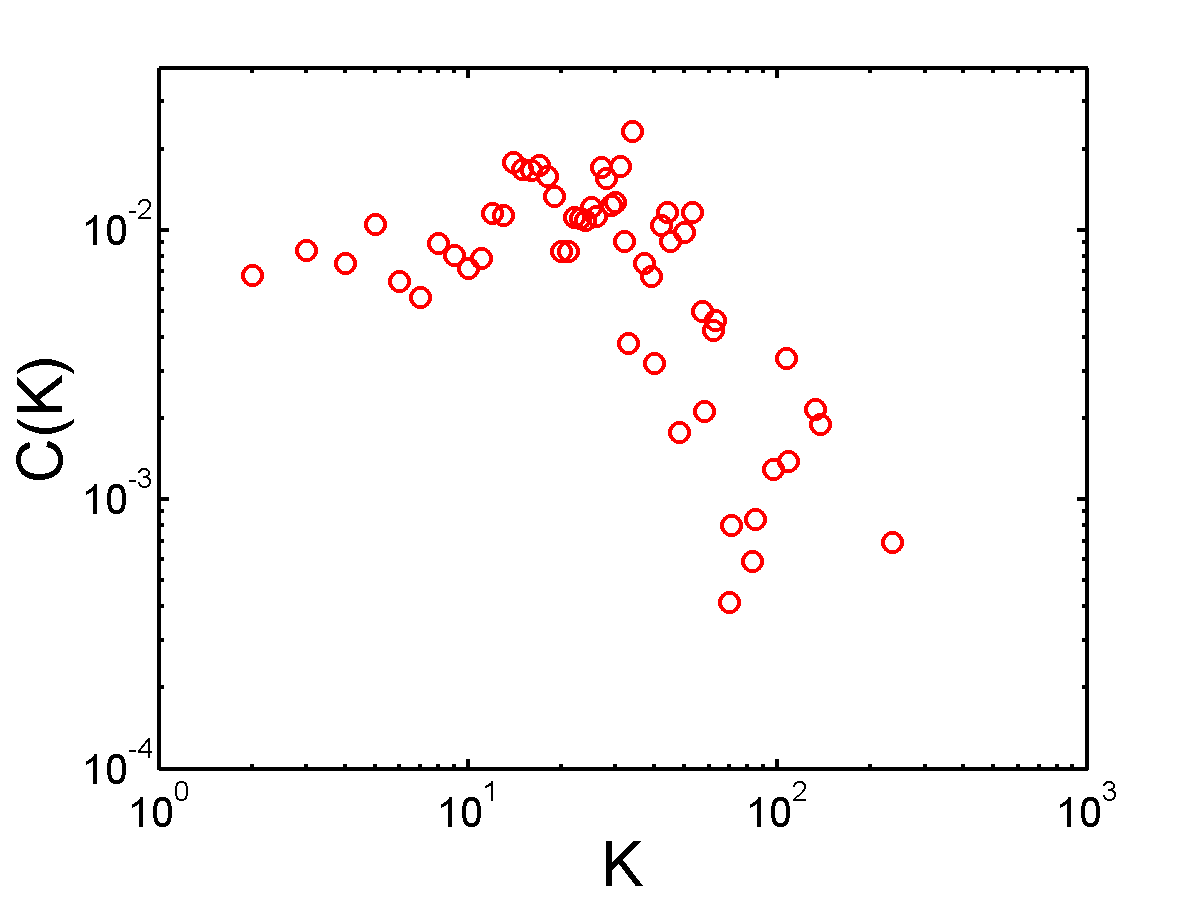


**Supplementary Figure 7.** The distribution of clustering coefficient for a random graph with the same degree correlation as PIN.
